# Evolving treatment strategies in Crohn’s disease

**DOI:** 10.1093/gastro/goad020

**Published:** 2023-04-19

**Authors:** Bo Shen

**Affiliations:** Center for Inflammatory Bowel Diseases, Columbia University Irving Medical Center/NewYork Presbyterian Hospital, New York, NY, USA

The natural history of Crohn’s disease (CD) dictates that patients require long-term medical therapy for induction and maintenance to relieve their symptoms, improve health-related quality of life, prevent tissue damage, lessen the risk for neoplasia, and reduce hospitalization and surgery. For the past quarter of a century, medical management of CD has evolved from corticosteroids for induction and immunomodulators for maintenance to antitumor necrosis factor, anti-integrin, and anti-interleukin biological agents and, in the near future, small molecule agents, for both induction and maintenance. Despite advances in medical therapy, the majority of patients with CD would eventually require surgery with incision and drainage of abscesses and fistulas, bowel resection and anastomosis, strictureplasty, or fecal diversion. Surgery will reset the clock for the disease course of CD and most patients require post-operative medical therapy to prevent disease recurrence. Colonoscopy plays a key role in disease monitoring and assessment of treatment response before and after surgery. Endoscopy also plays an important role in the treatment of CD- or its surgery-associated structural complications, such as primary or anastomotic strictures, abscesses, and fistulas.

In this Special Issue, we were able to assemble a panel of experts in medical, endoscopic, and surgical inflammatory bowel disease and to deliver seven articles based on current literature and authors’ experience and expertise [1–7]. The scope of this Special Issue covers medical (including the special setting of pregnancy), endoscopic, and surgical management of CD. We hope that the contents of the articles help to guide pre- and post-operative management strategies for practicing gastroenterologists, inflammatory bowel disease specialists, colorectal surgeons, students, trainees, and advanced care providers.
